# Rituximab used for simultaneous treatment of PR3‐ANCA positive vasculitis associated with rheumatoid arthritis: A case report

**DOI:** 10.1002/ccr3.3258

**Published:** 2020-08-26

**Authors:** Akihiro Yamada, Ayuko Sogabe, Yasuaki Okuda

**Affiliations:** ^1^ Center for Rheumatic Diseases Dohgo Spa Hospital Matsuyama Japan

**Keywords:** PR3‐ANCA positive vasculitis, rheumatoid arthritis, rituximab

## Abstract

We treated PR3‐ANCA positive vasculitis in a patient diagnosed with rheumatoid arthritis using rituximab. Monoclonal antibody therapy can be used to simultaneous treat more than one collagen disease in such patients. This suggests that shared pathogenic pathways exist between different collagen diseases.

## Graphical abstract

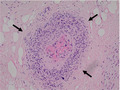

## 1 INTRODUCTION

We treated PR3‐ANCA positive vasculitis in an 80‐year‐old female patient who had bilateral ischemic peroneal nerve paralysis accompanied by rheumatoid arthritis with rituximab. Pathology showed mild vasculitis. Our findings could be an indication of a shared pathogenic pathway between PR3‐ANCA vasculitis and rheumatoid vasculitis.

PR3‐ANCA positive vasculitis is conventionally known as granulomatosis with polyangiitis (GPA) and autoimmune necrotization of the small vessels. The clinical manifestations of GPA usually involve the paranasal sinuses, lung, and kidney. We used monoclonal antibody(rituximab, a chimeric monoclonal antibody against the protein CD20 related to cell death: RTX) therapy to treat a patient with PR3‐ANCA positive vasculitis who presented with rare manifestations of ischemic peroneal nerve paralysis complicated with rheumatoid arthritis.

To our knowledge, successful RTX therapy for PR3‐positive vasculitis associated with rheumatoid arthritis at the same time has not been reported in the literature till date.

## 2 CASE PRESENTATION

**FIGURE 1:**
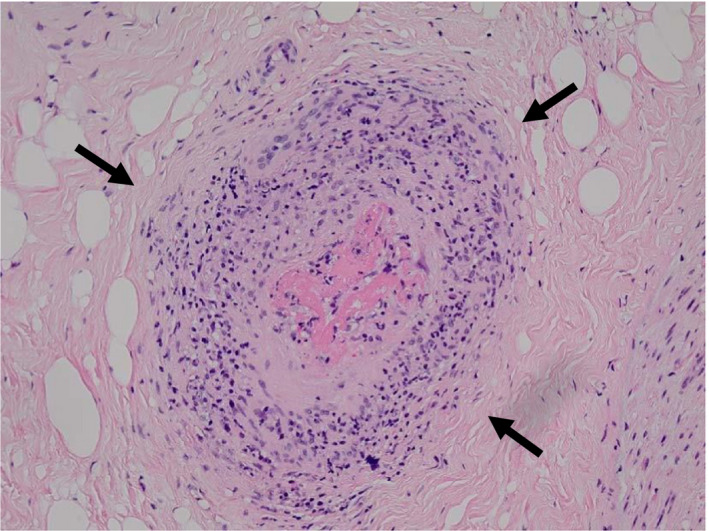
Biopsied specimen ×400 HE stain

An 80‐year‐old female was admitted to our hospital on 25 July 2014 with history of intermittent fever >39.0°C for a few months. In the previous hospital, she was diagnosed with rheumatoid arthritis (Stage V Class III, involving four swollen joints and seven tender joints). Intake of methotrexate was increased from 4 to 6 mg/wk. Nevertheless, her symptoms of morning stiffness, fever, and joint pains of bilateral shoulders, elbows, and wrists persisted. On admission to our hospital, computed tomography (CT) showed infiltrating shadows in the right lower bronchi and bronchiectasis, suggestive of a previous paragonimiasis infection. Paragonimiasis that had occurred during her childhood had completely resolved with treatment. Initially, a diagnosis of bronchiolitis owing to bacterial infection was suspected, for which, antibiotic therapy with tazobactam and piperacillin was administered. However, her symptoms did not improve. Several days after admission to our hospital, she had one episode of slight bleeding from the right nasal cavity. The antibody titers of myeloperoxidase‐3 had rapidly elevated and hence, we suspected GPA (Table 1). On 1 September 2014, we biopsied a vasculitic lesion from the right peroneal nerve which was causing paralysis of the corresponding limb with disability of dorsal flexion due to vasculitic ischemia. She still had slight nasal bleeding, intermittent fever, weight loss, and polyneuritis of the lower extremities. Pathological examination showed pauci‐immune vasculitis and granuloma formed on the outer side of the adventitia of the vasculitic lesion leading to the diagnosis of PR3‐ANCA positive vasculitis (Figure 1) clinically.^1^

**Table 1:** Laboratory data on admission

CBC		Immunology	
WBC	3000/µL	ESR	83 mm/h
Neu.	64%	CRP	1.76 mg/dL
Lym.	29%	SAA	64.3 µg/mL
Mono.	4%	MMP‐3	105 ng/mL
Eos.	0.0%	IgG	1876 mg/dL
Baso.	0.0%	IgA	433 mg/dL
RBC	330 × 10^4^/µL	IgM	201 mg/dL
Hb	8.9 g/dL	C3	31 mg/dL
Ht	27.3%	C4	2 mg/dL
Plt	18.6 × 10^4^/µL	CH50	8 U/mL
		ANA	×80homo, speckled
Biochemistry		ds‐DNA IgG < 10 IU/mL	
TP	6.1 mg/dL	RF	1297 IU/mL
Alb	2.5 mg/dL	IgG RF 4.0	
BUN	12.2 mg/dL	Anti‐CCP	1155.5 U/mL
Cr	0.37 mg/dL	Sm Ab	7.0 U/mL
AST	27 IU/L	IC	1.5 µg/mL
ALT	8 IU/L	CL‐Ab(IgG)	8 U/mL
ALP	174 IU/L	CL‐β2GPIAb	1.2 U/mL
γGTP	16 IU/L	PR3‐ANCA170 U/mL	
LDH	203 IU/L	MPO‐ANCA < 1.0 U/mL	
Na	141 mEq/L	IL‐6	19.4 pg/mL
K	3.1 mEq/L	sIL2‐r	1580 U/mL
Cl	106 mEq/L	EBvirus DNA <2.0 × 10	
Ca	7.6 mg/dL	T‐spot negative	
Fe	18 µg/dL	MAC Ab negative	
Ferritin	318 ng/mL	HbsAg negative	
T.Chol	115 mg/dL	HbsAb negative	
HDL‐C	32 mg/dL	HbcAb negative	
TG	92 mg/dL	Imaging	
LDL‐C	64 mg/dL	Scintigram PET‐CT no abnormal uptake	
		CT bronchiectasia in the right lung	

According to the therapeutic guidelines in Japan for ANCA‐associated vasculitides, 0.3‐0.6 mg/kg/d of prednisolone was initiated, but the patient did not respond. We subsequently attempted treatment with RTX (once weekly dose at 375 mg/m^2^/time, with four doses as loading therapy).

**FIGURE 2:**
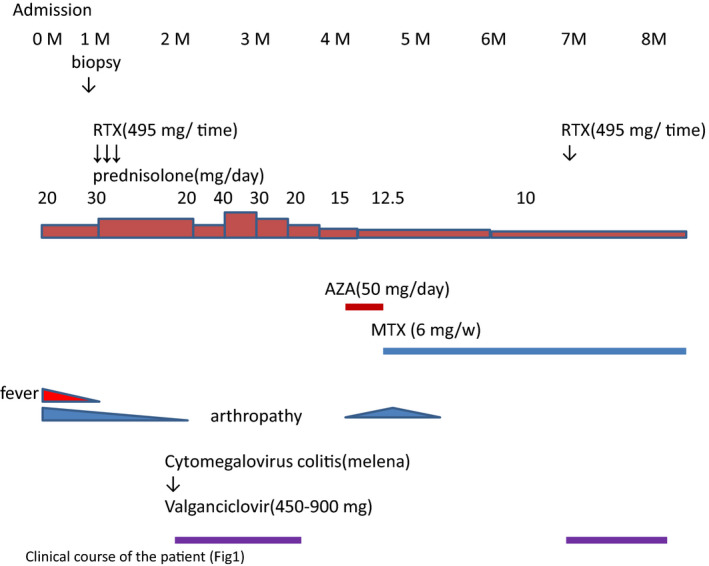
Clinical course of the patient

On September 17, September 24 and October 1, the patient received 495 mg of RTX as standard loading therapy. Three days after third administration, she developed melena caused by cytomegalovirus colitis (cytomegalovirus antigen [C7HRP] positive). Valgancyclovir was prescribed and the patient recovered quickly. From February 19 onwards, 495 mg of RTX was prescribed as maintenance therapy along with valgancyclovir (Figure 2). Following the loading therapy, the patient's fever caused by ANCA‐associated vasculitis and inflamed joints because of rheumatoid arthritis resolved. Four months after admission, her rheumatoid arthritis flared acutely, and we restarted methotrexate (6 mg/wk) to the treatment protocol. During hospitalization, she also underwent physical therapy with supportive ankle devices. Three years after RTX therapy, her peroneal nerve paralysis steadily recovered from a score of 1/5 on the manual muscle test to 3/5, defined as fair.

## 3 DISCUSSION

Etiologically, the HLA alleles in GPA have been reported as HLADPB1 *04:01.^2^, ^3^ In the present case, our patient was found to have HLADPB1 *04:02:01 and *05:01, which were unique to this patient.

No vasculitic lesions were observed on the CT in the nasal and maxillary sinuses or in the lung field, despite the symptoms of nasal bleeding, ruling out respiratory involvement. Urinalysis showed no proteinuria or hematuria (Table 1). Prior to the present course of hospitalization, the patient had intermittent fever for a few months and had suffered weight loss from about 50 to 40 kg over 6 months. She was aware of a paralytic disability of dorsal flexion of both ankles. Pathological examination of the biopsied specimen from the affected peroneal artery revealed thrombotic or apparent granulomatous tissue in the adventitia, suggestive of possible GPA. However, typically, in cases of GPA, granulomatous tissue is observed in the elastic lamina media. The titer of PR‐3 ANCA was high in our patient (170 U/mL), and hence, we diagnosed PR3‐ANCA positive vasculitis associated with rheumatoid arthritis and paralysis of both lower legs.

Immunohistopathological examination showed pauci‐immune vasculitis, with invasion of CD3 positive T cells and CD 68 positive or CD163 positive macrophages, and CD138 positive plasma cells present on the outer aspect of the adventitia. C3 staining was slightly positive.

Differentiating between the histological findings of GPA and rheumatoid vasculitis is challenging. On serological examination, the level of circulating immune complexes, von Willebrand factor, ICAM‐1, VCAM, E‐selectin, fibronectin, IgM RF, IgG RF, and IgA RF is lower in rheumatoid vasculitis than in rheumatoid arthritis.^4^ In the present case, immune complexes and IgG RF were not found on admission (Table 1).

Rheumatoid vasculitis is an important different diagnosis of ANCA‐associated vasculitis. Hypocomplementemia is commonly found in GPA, rheumatoid vasculitis, and systemic lupus erythematosus (SLE). However, in our case, the titer of anti‐PR3 ANCA was high, pauci‐immune vasculitis was noted, circulating immune complexes were absent, and she had no symptoms suggestive of SLE. Therefore, we diagnosed granulomatosis with polyangiitis complicated by rheumatoid arthritis.

**FIGURE 3:**
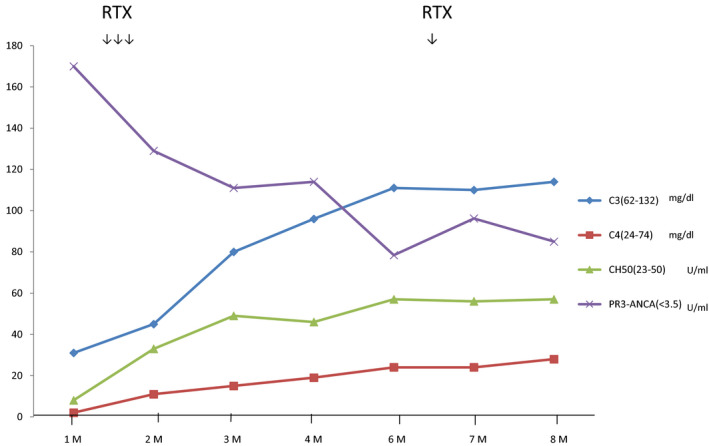
Clinical course GPA‐related marker

In the present case, the fever resolved after taking the first RTX infusion. The titer of anti‐PR3‐ANCA decreased from 142 to 78.4 U/mL in 4 months. We then added a standard dose of RTX every 6 months as maintenance therapy.^5^ The titers of anti‐PR3‐ANCA gradually reduced with maintenance RTX therapy to 85 U/mL at around 6 months following the first dose. Hypocomplementemia gradually improved and complement C3 levels returned to normal on November 18. C4 recovered slowly (2nd Feb) (Figure 3). GPA is known to activate an alternative pathway of complements.^6^ ANCA‐associated renal lesions are also related to the complement pathway,^7^ and therefore, GPA might reflect the level of C3.

**FIGURE 4:**
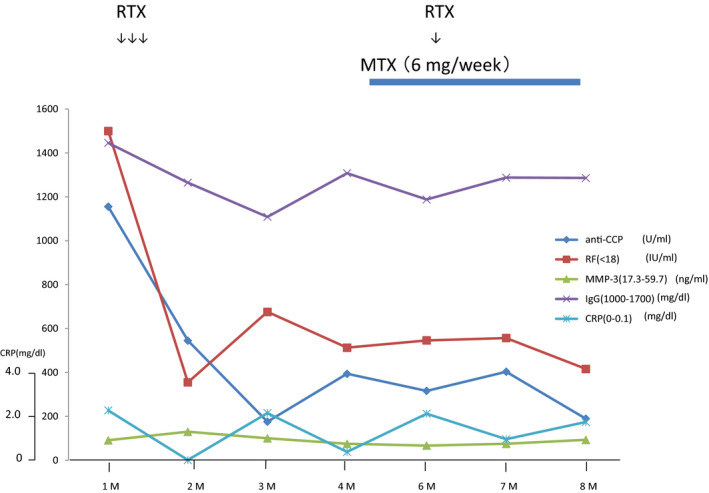
Clinical course RA‐related marker

Clinical symptoms of rheumatoid arthritis improved in this patient following loading therapy with RTX (Figure 4). The neurological recovery with standard maintenance therapy using RTX for GPA is not well established. Neurological deficits of polymononeuritis improved after the vasculitis resolved. ANCA‐associated vasculitis (AAV) often occurs with significant delay from the first rheumatological manifestations.^8^ The underlying mechanism of complication of AAV with RA is genetic disposition, and the involved points include the HLA region, PTPN22,^9^, ^10^, ^11^, ^12^ and polymorphism of uteroglobin.^13^

There is a high risk of developing lymphoproliferative disorders in general population with or without reactivation of EB virus on treatment with methotrexate.^14^ In our patient, no EB virus activation or abnormal accumulation of radioisotope on PET‐CT scans was noted on admission.

Over 6 months of RTX therapy, the granulomatosis of polyangiitis improved slowly and the complement levels normalized. In patients with MPO‐ANCA‐related nephropathy, low levels of C3 indicated poor prognosis.^15^ In our case, the vasculitis was mild and was localized to the lower legs, which was atypical. If we monitored the natural course of this patient, symptoms involving the nose, lung, and kidney could occur afterward. In a previous study, Federico et al reported the Birmingham Vasculitis Activity Score (BVAS) to be an excellent tool for assessing the in‐ICU mortality risk of patients with systemic vasculitis.^16^ In case of senile patients, adverse event of RTX is more important with respect to mortality than BVAS assessment. A marker that is more sensitive than MPO‐ANCA is required for granulomatosis of polyangiitis. As PR3‐ANCA positive vasculitis improved with RTX therapy, serological data reflecting the activity of rheumatoid arthritis improved as well, especially during the loading phase of therapy.^17^

## 4 CONCLUSION

RTX therapy was used to treat PR3‐ANCA positive vasculitis in an elderly patient with concomitant rheumatoid arthritis. Both collagen diseases simultaneously improved on treatment with the same drug. Our findings may be suggestive of a shared pathogenic pathway of PR3‐ANCA vasculitis and rheumatoid arthritis.

## CONFLICT OF INTEREST

None.

## AUTHOR CONTRIBUTIONS

YA, SA, and OY: were involved in care of the patient and collected clinical information and blood samples. YA and OY: coordinated this study and wrote the paper.
